# Phytochemical Profiling, Antioxidant Activity, and Protective Effect against H_2_O_2_-Induced Oxidative Stress of *Carlina vulgaris* Extract

**DOI:** 10.3390/molecules28145422

**Published:** 2023-07-15

**Authors:** Ireneusz Sowa, Jarosław Mołdoch, Sławomir Dresler, Tomasz Kubrak, Agata Soluch, Dariusz Szczepanek, Maciej Strzemski, Roman Paduch, Magdalena Wójciak

**Affiliations:** 1 Department of Analytical Chemistry, Medical University of Lublin, Chodźki 4a, 20-093 Lublin, Poland; slawomirdresler@umlub.pl (S.D.); maciej.strzemski@poczta.onet.pl (M.S.); 2 Department of Biochemistry and Crop Quality, Institute of Soil Science and Plant Cultivation, State Research Institute, 24-100 Puławy, Poland; jmoldoch@iung.pulawy.pl (J.M.); agata.soluch@iung.pulawy.pl (A.S.); 3 Department of Plant Physiology and Biophysics, Institute of Biological Science, Maria Curie-Skłodowska University, Akademicka 19, 20-033 Lublin, Poland; 4 Department of Biochemistry and General Chemistry, Institute of Medical Studies, Medical College, Rzeszów University, 35-310 Rzeszów, Poland; tkubrak@ur.edu.pl; 5 Chair and Department of Neurosurgery and Paediatric Neurosurgery, Medical University of Lublin, 20-090 Lublin, Poland; dariusz.szczepanek@umlub.pl; 6 Department of Virology and Immunology, Institute of Biological Sciences, Faculty of Biology and Biotechnology, Maria Curie-Skłodowska University, 19 Akademicka Street, 20-033 Lublin, Poland; roman.paduch@mail.umcs.pl

**Keywords:** polyphenols, flavonoid C-glycosides, antioxidant, H_2_O_2_-induced stress, human skin fibroblast

## Abstract

*Carlina vulgaris* is a little-understood plant with unexplored biological potential, and the papers regarding its chemical composition are scarce. In our study, for the first time, the phytochemical profile of the plant, focusing on polar metabolites, was established using modern chromatographic techniques including LC-HRMS-QTOF-CAD, UHPLC-PDA-MS. Phytochemical analysis revealed that the species is a rich source of polyphenolic components, with the most abundant being chlorogenic acid and C-glycosides of luteolin, including carlinoside, orientin, isoorientin, and C-glycosides of apigenin, schaftoside, isoschaftoside, and vitexin. Furthermore, we assessed the impact of the polyphenolic-rich fraction of *C. vulgaris* extracts on human skin fibroblasts using the MTT and NR assays. It was found that the extract was non-toxic and exhibited potent antioxidant activity in the cells subjected to induced oxidative stress. Additionally, it effectively protected the cells against H_2_O_2_-induced cytotoxicity. Our study contributes to the general trend of searching for new phytotherapeutics with potential applications in pharmacy and medicine. The results indicate that further exploration of *C. vulgaris* species is worthwhile, as they can serve as valuable plant material for cosmetic use.

## 1. Introduction

The genus *Carlina*, belonging to the Asteraceae family, comprises over 30 species that can be found in Europe and Asia in their natural environment. Plants from the genus *Carlina* have been widely used in traditional medicine in many countries, including Spain, Italy, Hungary, Lithuania, Poland, and the Balkan countries, due to their high potential for medicinal purposes [1,2,3,4,5,6,7,8]. They have been applied to gastrointestinal dysfunctions such as gastritis and dyspepsia and as a cholagogic agent [2,3]. Furthermore, extracts from the herbs have been found to be useful in facilitating the healing of skin lesions, wounds, ulcerations, skin infections, rough skin, and swellings [1,9,10,11].

However, to date, most species from the genus are poorly understood in terms of both biological activity and phytochemistry. Currently, the most knowledge is available about *C. acaulis*, which is the subject of intensive research. Many scientific reports have confirmed its multidirectional biological activity, including antioxidant, antibacterial, antifungal, insecticidal, and anti-ulcer properties and cytotoxicity against a few types of cancer lines [12,13,14,15,16,17,18,19,20,21]. The plant has also been shown to be a rich source of valuable components including volatile compounds of essential oil, phenolic acids, and pentacyclic triterpenes [22,23,24,25]. These findings justify the traditional usage of *Carlina* species and demonstrate the enormous potential of the genus.

**Figure 1:**
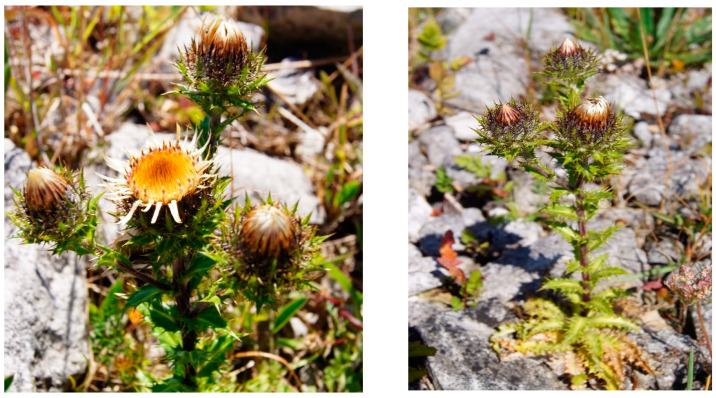
An example of *C. vulgaris* growing in its natural habitat.

*C. vulgaris* L. is one of the less studied species from the *Carlina* genus. However, due to its taxonomic affinity with *C. acaulis*, it could potentially serve as another source of metabolites with high biological activity. It is a monocarpic perennial plant widespread in central and western Europe, from the southern regions of the Iberian and Apennine Peninsulas to southern Sweden. It primarily colonizes calcareous grasslands [26]. The plant produces a stiff, erect, top-branching stem that can reach a height of up to 80 cm. The leaves are lanceolate, serrated, and strongly spiny. The inflorescence consists of one or several baskets, measuring 3.5–5 cm in diameter (Figure 1).

Despite a relatively detailed botanical description [27], there are limited available data on the phytochemistry of this plant. Strzemski et al. discovered pentacyclic triterpenes, including oleanolic and ursolic acid, lupeol, lupeol acetate, α and ß amyrins, and ß amyrin acetate in the inflorescences, green parts, and roots of *C. vulgaris*. [23]. Furthermore, both the aboveground parts and roots of *C. vulgaris* plants were found to be a rich source of chlorogenic acid, with the highest content observed in the leaves (approximately 3 mg/g) [28]. Polyacetylenes, with the most abundant carlina oxide (33.7%) and 13-methoxy carlina oxide (11.5%), were identified as the main components of the root essential oil from *C. vulgaris* [29].

The knowledge regarding the biological properties of *C. vulgaris* is also scarce. It has been found that the plant extracts display a free radical scavenging effect in ABTS and DPPH tests [28], and the essential oil shows antioxidant and antifungal activity against *Penicillium expansum* and *Aspergillus niger* [29]. Additionally, carlina oxide found in the oil exhibited antibacterial and antifungal activity against various strains of bacteria and fungi. It also demonstrated an antiparasitic effect against *Trypanosoma brucei* [30].

The gathered information indicates that it is worthwhile to investigate *C. vulgaris* in more detail. Therefore, the aim of our research was to address this issue and expand our understanding of this species. The main goal was to establish the phytochemical profile of the plant, focusing on polar metabolites, using modern chromatographic techniques including liquid chromatography with a quadrupole-time-of-flight high-resolution mass spectrometer (LC-HRMS-QTOF), a charged aerosol detector (CAD), and ultra-performance liquid chromatography (UHPLC) coupled to photodiode array detection (PDA) and mass spectrometry (MS). Furthermore, the antioxidant potential and protective effect against H_2_O_2_-induced oxidative stress were investigated in human skin fibroblasts (HSF), which could provide an explanation for the traditional application of *Carlina* plants in skin disorders.

## 2. Results

### 2.1. Plant Material, Phytochemical Profiling and Quantification of the Components

A total of 1.2 kg of the aboveground parts of the plants was obtained from field cultivation of *C. vulgaris*, which yielded 312 g of lyophilized material. A total of 57 g of dried extract was obtained after exhaustive extraction of 300 g of freeze-dried samples with 70% methanol (the methanolic extract of *C. vulgaris*—ECV).

**Figure 2:**
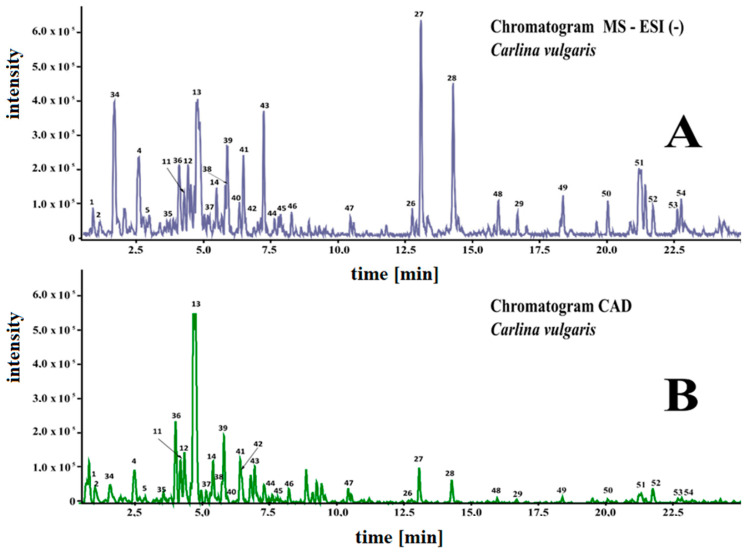
Chromatograms of extracts from *C. vulgaris* obtained using liquid chromatography with a quadrupole-time-of-flight high-resolution mass spectrometer (LC-HRMS-QTOF) and a charged aerosol detector (CAD). (**A**)—MS chromatogram (electrospray ionization—ESI); (**B**)—CAD chromatogram.

The phytochemical composition of the methanolic/water extract from the aboveground parts of *C. vulgaris* was determined using UHPLC-MS. The components were analyzed in negative and positive ionization modes and identified based on mass data (M-H), fragmentation patterns, and UV-Vis spectra (200–600 nm). Representative chromatograms are shown in Figure 2.

The results of qualitative analysis and quantification, expressed per gram of dried plant material, are shown in Table 1.

A total of fifteen different phenolic compounds were identified in the *C. vulgaris* extract. Among the phenolic acids, chlorogenic acids, including 3-caffeoquinic and 5-caffeoquinic (neochlorogenic), were the most abundant, with contents of 6.90 and 1.02 mg/g of dry weight, respectively. This was followed by densifloside (4.98 mg/g) and a low amount of dihydroxybenzoic acid (0.09 mg/g). A total of 0.23 mg/g of quinic acid (cyclohexanecarboxylic acid) was also found in the aboveground part of *C. vulgaris*. Flavonoids were mainly represented by C-glycosides of luteolin and apigenin. Vitexin (apigenin-8-C-glucoside), isoschaftoside (apigenin-6-C-arabinoside-8-C-glucoside), and carlinoside (luteolin 6-C-glucoside-8-C-arabinoside) were detected at the highest amounts with mean contents of 4.0 mg/g, 3.9 mg/g, and 3.1 mg/g, respectively. Orientin (luteolin-8-C-glucoside), schaftoside (Apigenin-6-C-glucoside-8-C-arabinoside), and isoorientin (luteolin-6-C-glucoside) were present in amounts ranging from 1.3 mg/g to 1.9 mg/g. Additionally, a small quantity (0.05 mg/g) of apigenin di-C-arabinoside was identified. Rutin (quercetin-3-O-rutinoside,1.30 mg/g) and taxifolin (dihydroquercetin, 0.06 mg/g) were also found in the extract. Furthermore, several lipidic constituents including linoleic and octadecenoic acid derivatives and two amino acids (L-tryptophan and methyltryptophan) were present in methanol/water extract from the plant.

### 2.2. Fractionation and Phytochemical Characterization of the Fractions

**Figure 3:**
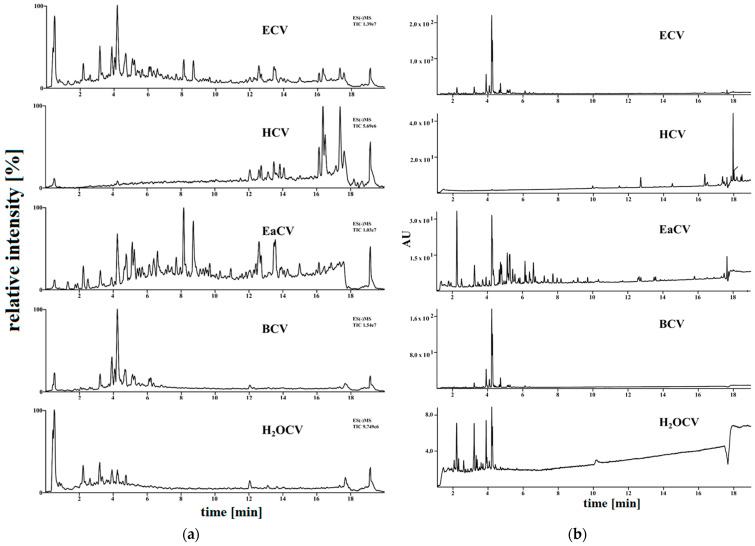
The chromatograms obtained from ultra-performance liquid chromatography with mass spectrometry and electrospray ionization UHPLC-ESI-MS(−) chromatograms (**a**) and the UHPLC with photodiode detector—PDA (254 nm) (**b**). Fractions obtained through liquid–liquid extraction from the extract of *C. vulgaris*. ECV—methanol extract, HCV—hexane fraction, EaCV—acetate fraction, BCV—butanol fraction, H_2_OCV—water fraction.

The methanolic extract was fractionated using solvents with different polarity to obtain a polyphenolic-rich fraction. The yields of extracts obtained from subsequent fractionation of ECV with hexane (HCV), ethyl acetate (EaCV), and n-butanol (BCV) were 1.55 g, 0.7 g, and 1.45 g, respectively. The remaining 53.3 g of solid residues were dissolved in water (H_2_OCV). The UHPLC-MS and PDA chromatograms of the fractions are shown in the Figure 3, and the results of quantitative analysis of the components in the fractions, expressed per gram of dried extract, are summarized in Table 2.

The results of the chromatographic analysis showed that the ethyl acetate fraction (EaCV) contained the highest content of chlorogenic acids (181 mg/g of dried fraction), densifloside (113.73 mg/g), C-glycosides of apigenin (174.7 mg/g), followed by C-glycosides of luteolin (57.7 mg/g), and rutin (23.9 mg/g). The butanol fraction (BCV) contained the highest content of orientin and isoorientin (57.5 mg/g), a low amount of densifloside (7.9 mg/g), and dihydroxybenzoic acid (5.8 mg/g), as well as trace amounts of polyphenolic compounds. No phenolic constituents were identified in HCV, which is not surprising because they are polar compounds poorly dissolved in nonpolar solvents such as hexane.

### 2.3. Antioxidant Assay

In order to assess the antioxidant properties of the tested samples, two different tests, namely DPPH and FRAP, were used. The free radical scavenging capacity (DPPH assays) was expressed as Trolox equivalent. Ferric reducing antioxidant power (FRAP) was calculated as equivalent of ascorbic acid. The results for the DPPH and FRAP assays are shown in Table 3.

As can be seen, EaCV exhibited the highest free radical scavenging activity in the DPPH test and the highest ferric reducing ability. It was followed by BCV, H_2_OCV, and HCV in terms of antioxidant potential. These findings were consistent with the results of quantitative analysis, as EaCV showed significantly higher content of polyphenolic compounds from phenolic acids and flavonoid classes.

### 2.4. Antioxidant Assay Using Human Cell Fibroblasts

Given that EaCV is the richest in polyphenolic compounds fraction with the highest antioxidant potential, it was selected for further antioxidant testing using HSF cells.

#### 2.4.1. Cell Viability Assay

**Figure 4:**
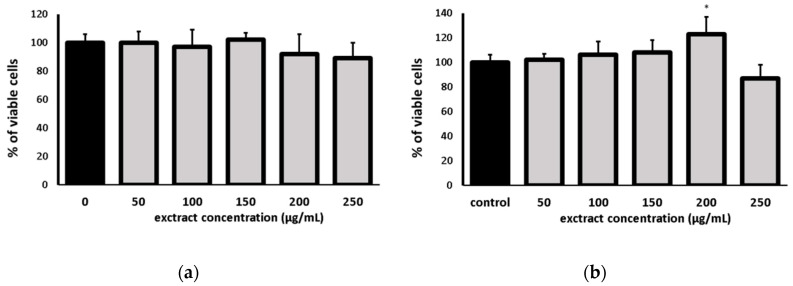
Effect of the different concentrations of ethyl acetate fraction (EaCV), obtained from methanol/water extract of *Carlina vulgaris* on cell viability determined by the NR (**a**) and MTT (**b**) assay, expressed as a % of control (0.5% of DMSO in medium). The data are means (*n* = 3) ± SD. One-way ANOVA followed by Dunnett’s post hoc test; the differences were considered significant at *p* < 0.05. * indicates statistically significant difference.

To determine the non-toxic concentration of EaCV extract, the cytotoxicity on fibroblast cells was evaluated using two complementary assays: the neutral red (NR) test and the MTT test (Figure 4). The NR test assesses the stability of cell membranes based on the uptake of the dye via active transport and its accumulation in lysosomes of viable cells. In turn, the MTT test demonstrates the impact on cellular metabolism, specifically the activity of NAD(P)H-dependent cellular oxidoreductases.

The NR assay demonstrated that the extract did not have a negative impact on cell viability and did not decrease, in a statistically significant manner, the number of living cells in the culture within the tested concentrations range. Furthermore, at a concentration of 200 µg/mL, it even slightly stimulated cell metabolism.

#### 2.4.2. Protective Effect of Extract on H_2_O_2_-Induced Cytotoxicity

**Figure 5:**
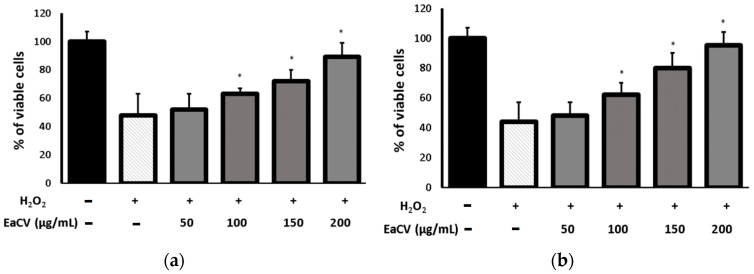
Effect of different concentrations of ethyl acetate fraction (EaCV), obtained from the methanol/water extract of *Carlina vulgaris*, on H_2_O_2_-treated cells evaluated in terms of (**a**) cell viability (NR) and (**b**) cellular metabolism (MTT). Cells were pretreated with extract at different concentrations prior to the H_2_O_2_ exposure. The results are expressed as a percentage of the control (0.5% DMSO). The data are means ± SD (*n* = 3). * indicates a statistically significant difference (*p* < 0.05) versus H_2_O_2_-treated cells assessed using one-way ANOVA followed by Dunnett’s multiple comparison post hoc test.

The protective effect of the extract against H_2_O_2_-induced cytotoxicity was investigated by assessing cell viability and cell metabolism. The cells were pretreated with increasing concentrations of the extract and then exposed to H_2_O_2_. The results are presented in Figure 5.

It was observed that H_2_O_2_ treatment significantly reduced the percentage of viable cells and suppressed the enzymatic activity of the cells compared to the control. However, when the extract was added 30 min prior to H_2_O_2_ exposure, at a concentration of 200 µg/mL, it protected the cells from these negative effects.

#### 2.4.3. Antioxidant Activity of the Extract in H_2_O_2_-Induced Oxidative Stress

**Figure 6:**
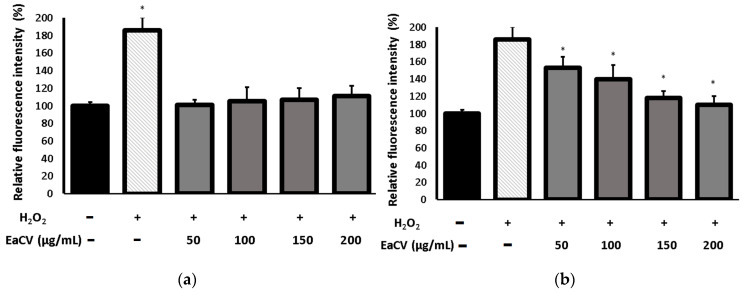
Relative fluorescence of 2′,7′-dichlorodihydrofluorescein (DCF) in human skin fibroblast cells calculated as a percentage in comparison with untreated control cells. (**a**)—the cells were treated with H_2_O_2_ or different concentrations of ethyl acetate fraction (EaCV), obtained from the methanol/water extract of *Carlina vulgaris*. * indicates a statistically significant difference (*p* < 0.05) versus untreated controls. (**b**)—the cells were pretreated with EaCV prior to the H_2_O_2_ exposure. * indicates a statistically significant difference (*p* < 0.05) versus the H_2_O_2_-treated cells. The data are means ± SD (*n* = 3). One-way ANOVA followed by Dunnett’s multiple comparison post hoc test.

The assay aimed to find out whether the protective effect of the ethyl acetate fraction (EaCV) on human skin fibroblasts exposed on H_2_O_2_ induced oxidative stress is a result of its impact on oxidative imbalance. To evaluate the protective action of EaCV against disturbance in the oxidative balance, the influence on the intracellular production of reactive oxygen species was determined using the H_2_DCFDA test. The results are shown in Figure 6.

As can be seen, H_2_O_2_ strongly induced oxidative stress and the level of ROS significantly increased (up to 184%) compared to the control. In turn, the EaCV extract did not affect the oxidative balance in a statistically significant manner (Figure 6a). Pretreatment with the EaCV suppressed ROS production in H_2_O_2_-stimulated cells in a concentration-dependent manner.

## 3. Discussion

*Carlina vulgaris* is a little-understood plant with unexplored biological potential, and papers regarding its chemical composition are scarce. To date, only a few triterpenic compounds and chlorogenic acid have been identified in the aboveground parts and roots of *C. vulgaris* [23,28]. In turn, analysis of the root essential oil revealed the presence of thirteen volatile components, with the prevalence of polyacetylenes, carlina oxide, and its 13-methoxy derivative [29].

Our study shows that the aboveground parts of *C. vulgaris* are a rich source of polyphenolic components from the phenolic acids and flavonoids classes, with the most abundant being flavonoid C-glycosides. In contrast to the most widespread O-glycosides, in which the sugar is linked to the aglycone via an oxygen atom, the characteristic feature of C-glycosides is the attachment of sugar moieties directly to the flavonoid backbone through C-C covalent bonds. This makes their structure more stable than O-glycosides, and therefore, C-glycosides differ in pharmacokinetics and biological activities [31,32,33].

C-glycosides are relatively less studied flavonoid derivatives; however, recent literature data have shown that they possess many beneficial effects and health-promoting properties, including antioxidant, antibacterial, antiviral, anti-diabetic, anti-inflammatory, neuroprotective, and antihypertensive potential [31,34,35,36,37,38]. Our investigation for the first time reveals that *C. vulgaris* contains a high amount of C-glycosides of luteolin, including carlinoside, orientin, isoorientin, and C-glycosides of apigenin, schaftoside, isoschaftoside, and vitexin. Some of these C-glycosides, namely orientin, homoorientin, vitexin, and isoschaftoside, were previously detected in another *Carlina* species—*C. acaulis* [39,40]. However, quantitative data regarding their amounts were lacking.

Fractionation of the methanolic extract with ethyl acetate yielded a polyphenolic-rich fraction (EaCV) which showed a protective effect against H_2_O_2_-induced oxidative stress 253 and prevented H_2_O_2_-induced cytotoxicity. It can be supposed that the effect was related with polyphenolic compounds which are known for their potent antioxidant properties and beneficial effects on cell metabolism [41,42]. For example, it has been evidenced that chlorogenic acid (CA), an abundant component of *C. vulgaris*, stimulates the proliferation of skin fibroblasts and keratinocytes, and it contributes to the production of matrix proteins. It also exhibits a strong protective effect against oxidative stress [43,44,45,46]. Free radical scavenging effects and protective activities against reactive oxygen species (ROS) have also been found for many flavonoid C-glycosides in both in vitro and in vivo assays. It has been reported that vitexin acts as an effective radical scavenger and protects against lipid peroxidation and other oxidative damages in various oxidative stress-related diseases [47]. Additionally, orientin and vitexin have been shown to improve the endogenous antioxidant status in the organism and increase levels of superoxide dismutase, catalase, and glutathione peroxidase in the serum [48]. Furthermore, it has been demonstrated that isoschaftoside, isovitexin, vitexin, and orientin protect against lipopolysaccharide-stimulated inflammation [37,49].

Plant products with high antioxidant activity are strongly desirable components of skin care products. It is well known that oxidative stress plays a significant role in skin aging and favors the development of skin-related disorders. Excessive exposure to reactive oxygen species disturbs cellular redox balance and consequently leads to the damage and dysfunction of the cells. ROS may modify the micro-environment of the skin through modulation of the extracellular matrix and affect the matrix metalloproteinases responsible for tissue remodeling, which leads to structural and functional alterations in skin, including collagen fragmentation and disorganization of collagen fibers. Furthermore, ROS-related cell damage increases the level of pro-inflammatory cytokines and promotes a chronic inflammatory state [50,51,52]. All these processes accelerate skin aging.

Our study has revealed that *C. vulgaris* is rich in polyphenolic compounds with health-promoting properties. Therefore, it is worthwhile to continue investigating this plant and exploring other potential courses of action.

## 4. Materials and Methods

### 4.1. Reagents and Standards

Analytical standards of phenolic compounds and reagents, including 2,2-diphenyl-1-picrylhydrazyl (DPPH), o-phenanthroline and ferric chloride (FRAP reagent), were purchased in Fluka (Sigma-Aldrich Co., St. Louis, MO, USA).

LC-MS-grade methanol, acetonitrile, and formic acid were purchased from Merck KGaA (Darmstadt, Germany). The other solvents were analytical-grade (Merck).

Water was deionized and purified using Ultrapure Millipore Direct-Q^®^ 3UV-R (Merck, KGaA, Darmstadt, Germany).

### 4.2. Plant Material

The seedlings of *Carlina vulgaris* plants were obtained from seeds collected from plants growing in the UMCS Botanical Garden in Lublin (voucher specimen no. 9/2009S). The cultivation lasted for 2 years (2018–2019) in the field at the Botanical Garden [53]. No fertilization was used. The plants were collected in August 2019 (during the flowering phase). The plant material was rinsed with running water, dried, freeze-dried and stored at a temperature of −20 °C until further processing.

### 4.3. Extraction and Fractionation

The aboveground parts of the plants were ground, and 300 g of material was exhaustively extracted with methanol and 70% methanol (3 × 5 L and 3 × 1.5 L for 15 min each) using an ultrasonic bath. The extracts (ECV) were combined, centrifuged at 8000 rpm, filtered, concentrated using a vacuum evaporator, frozen, and subjected to freeze-drying.

The freeze-dried ECV was suspended in 300 mL of MeOH and subjected to liquid–liquid extractions with n-hexane (5 × 100 mL) followed by ethyl acetate (5 × 100 mL). The residue was evaporated to dryness and suspended in water, then extracted with n-butanol (5 × 100 mL). The residue after extraction represented the H_2_OCV fraction. The fractions were concentrated using a vacuum evaporator, frozen, and freeze-dried.

### 4.4. Chromatographic Analysis (UHPLC–HR/QTOF/MS–CAD–PDA)

The lyophilized samples were reconstituted in 50% MeOH in Milli-Q water with the addition of 5% dimethyl sulfoxide (DMSO), centrifuged, and filtered.

The chromatographic analyses of the extracts were performed using an ultra-high-performance liquid chromatography (UHPLC) system coupled with a charged aerosol detector (CAD) and a high-resolution/quadrupole-time-of-flight mass spectrometer (HR/QTOF/MS Impact II) employing electrospray ionization (ESI). The chromatographic separation was conducted on a BEH C18 column (2.1 × 150 mm, 1.7 µm; Waters) at a temperature of 40 °C. A linear gradient elution was applied with a flow rate of 0.5 mL/min, using solvent B (acetonitrile-0.1% formic acid (FA)) in solvent A (H_2_O-0.1% FA), ranging from 2% B to 80% B, over 30 min. UV spectra of the compounds were recorded in the range of 190–750 nm with a resolution of 3.6 nm. MS spectra were acquired in negative modes, scanning in the range of 80–2000 *m*/*z*. Nitrogen gas was used as the cone and spraying gas, with flow rates of 800 L/h and 100 L/h, respectively. The capillary voltage was set at −2.8 kV for negative mode. The cone voltage was −25 V and 45 V, respectively, the source temperature was 140 °C, and the spraying gas temperature was 350 °C. The data acquisition and processing were performed using Waters Mass Lynx software. Quantitative analyses were performed using an ultra-performance liquid chromatography system UPLC-PDA-ESI-MS (ACQUITY, Waters) coupled with a PDA detector and a triple quadrupole mass spectrometer (ACQUITY TQD, Waters). The chromatographic conditions are described above.

### 4.5. Antioxidant Activity

#### 4.5.1. DPPH Radical Scavenging Assay

The 2,2-diphenyl-1-picrylhydrazyl (DPPH) assay was carried out according to procedure published previously [54]. EsCV was dissolved, diluted, and mixed with a 4 mM methanolic DPPH solution. Absorbance was measured at the wavelength λ = 517 nm using a UV-VIS Filter Max 5 spectrophotometer (Thermo Fisher Scientific, Waltham, MA, USA). Water with a DPPH solution was used as a control.

#### 4.5.2. Ferric Ion Reducing Antioxidant Power (FRAP Assay)

The ferric-reducing activities of the samples were determined according to the method described by Sowa et al. [55] with some modifications. The sample (15 μL) was mixed with fresh FRAP reagent (350 μL). The 300 mM acetate buffer pH 3.6, 10 mM TPTZ in 40 mM HCl, and 20 mM FeCl_3_·6H2O were mixed according to 10:1:1 as FRAP reagent and reacted in the dark for 5 min. The absorbance was measured at λ = 593 nm.

### 4.6. Cell Culture and Experimental Design

Human skin fibroblast cells line (ATCC^®^ CRL-2522™) were from the American Type Culture Collection (Manassas, VA, USA). The cells were grown in Dulbecco’s modification of Eagle’s medium (DMEM, Biological Industries, Cromwell, CO, USA) supplemented with sodium pyruvate, L-glutamine, 10% fetal bovine serum (Gibco, Waltham, MA, USA), glucose (4.5 g/L), and 1% antibiotics (100 U/mL of penicillin and 1000 of µg/mL streptomycin, Gibco). Cultured cells were kept at 37 °C in a humidified atmosphere of 95% air and 5% carbon dioxide. When the cells obtained the required confluence, the medium was removed, and the cells were rinsed twice with sterile phosphate-buffered saline (PBS, Gibco, Waltham, MA, USA). The confluent layer was trypsinized (0.25% Trypsin/EDTA, Gibco) and placed in fresh medium.

For cytotoxicity assay, the cells (1 × 10^5^ cells/mL) were plated in 96-well flat-bottom plates, incubated for 24 h at 37 °C, and then treated with the EaCV extract for 24 h.

For antioxidant assay, the cells (1 × 10^5^ cells/mL) were seeded on the well bottom in 96-well plates. After 24 h of incubation at 37 °C, the cells were pretreated with the EaCV extract and, after 30 min, H_2_O_2_ (250 μM) was added to the medium to induce oxidative stress [56]. Stock solution of the EaCV extract was prepared using DMSO/culture medium (1:1) and appropriately diluted. The final concentration of DMSO did not exceed 0.5%. Cells treated with 0.5% of DMSO in culture medium was taken as control.

All experiments were performed in triplicates for each extract concentration and presented as a percentage of the control (100%).

### 4.7. Cell Viability Assay

#### 4.7.1. MTT Assay

After 24 h of incubation with the EaCV extract, a 3-(4,5-dimethylthiazole-2-yl)-2,5-diphenyltetrazolium bromide solution (MTT) at a concentration of 5 mg/mL (Sigma) was added to the cells (25 μL/well), followed by further incubation for 3 h. The insoluble formazan crystals were solubilized overnight in a mixture of 10% sodium dodecyl sulfate (SDS) in 0.01 M HCl. Absorbance was measured at 570 nm wavelength using an E-max Microplate Reader (Molecular Devices Corporation, Menlo Park, CA, USA).

#### 4.7.2. Neutral Red Uptake Assay

After 24 h incubation of the cells with the EaCV extract, the solution was removed from the wells, and the cells were 2 h incubated with a solution of a neutral red dye (40 μg/mL) at 37 °C. The cells were washed with phosphate-buffered saline (PBS), PBS was removed, and 150 µL of decolorizing buffer was added. The plates were shaken for 10 min, and the optical density (OD) of the eluted dye was measured at 540 nm using an E-max Microplate Reader (Molecular Devices Corporation, Menlo Park, CA, USA). The results are presented as the percentage of the amount of dye retained compared to the control cells (100%)

### 4.8. Analysis of Intracellular Reactive Oxygen Species

The generation of intracellular reactive oxygen species in the human fibroblasts was performed as previously described [57]. After 24 h of incubation, the medium was removed and replaced with 10 μM H_2_DCFDA (Sigma Aldrich), and the cells were incubated for 45 min at 37 °C. The fluorescence was measured after 90 min using a FilterMax F5 microplate reader (Thermo Fisher Scientific) at a maximum excitation of 485 nm and emission spectra of 530 nm.

### 4.9. Statistical Analysis

All analyses were carried out in triplicate. The results were analyzed using Statistic ver. 13.3 software. One-way ANOVA followed by Dunnett’s post hoc test was used. The values were expressed as the mean ± standard deviation (SD). The differences were considered significant at a *p*-value of ≤0.05.

## Figures and Tables

**Table 1:** Compounds found in the extract of aerial parts of *C. vulgaris*. Amount was expressed in milligrams per gram of dry weight (mg/g d.w).

nr	RT (min)	M/Z	MS2	Ion Formula [M/Z-H]	Δppm	Identified	Amount (mg/g d.w)
1	0.8	191.055878	191, 135	C_7_H_11_O_6_	1.2	quinic acid	0.23 ± 0.01
2	1.3	153.019395	153, 109	C_7_H_5_O_4_	−0.4	di-hydroxybenzoic acid	0.09 ± 0.01
34	2.0	203.082571	203, 116	C_11_H_11_N_2_O_2_	0.2	L-tryptophan	
4	2.5	353.087769	353, 351, 191, 133	C_16_H_17_O_9_	0.1	neochorogenic acid	1.02 ± 0.01
5	2.6	353.087764	353, 191	C_16_H_17_O_9_	0.1	chlorogenic acid	6.90 ± 0.01
35	2.9	215.082690	215, 171, 142, 116	C_12_H_11_N_2_O_2_	−0.4	methyltryptophan	
36	4.0	579.136415	579, 489, 399, 369	C_26_H_27_O_15_	−1.5	carlinoside	3.10 ± 0.01
11	4.4	563.141367	563, 473, 443, 383, 353	C_26_H_27_O_14_	−1.3	schaftoside	1.59 ± 0.01
12	4.6	447.094105	447, 429, 357, 327, 297	C_21_H_19_O_11_	−1.8	orientin	1.86 ± 0.02
13	4.7	563.141444	563, 503, 473, 443, 383, 353	C_26_H_27_O_14_	−1.4	isoschaftoside	3.88 ± 0.01
37	5.1	303.051651	303, 285, 217, 125	C_15_H_11_O_7_	−2.1	taxifolin	0.06 ± 0.01
14	5.2	563.141926	563, 503, 473, 443, 383, 353	C_26_H_27_O_14_	−2.3	isoorientin	1.28 ± 0.01
38	5.4	609.147050	609, 300	C_27_H_29_O_16_	−1.5	rutin	1.30 ± 0.01
39	5.6	431.099416	431, 341, 311, 283	C_21_H_19_O_10_	−2.4	vitexin	4.02 ± 0.01
40	5.9	533.131166	533, 515, 473, 443, 383, 353	C_25_H_25_O_13_	−2.1	apigenin di-C arabinoside	0.05 ± 0.01
41	6.3	385.115218	385, 207, 177, 129	C_17_H_21_O_10_	−3.1	densifloside	4.98 ± 0.01
42	6.4	593.152523	593, 285	C_27_H_29_O_15_	−2.2	nicotiflorin	0.99 ± 0.01
43	7.2	187.098392	187, 169, 125	C_9_H_15_O_4_	−4.3	azelaic acid	
44	7.7	243.124940	243, 225, 199, 181, 163	C_12_H_19_O_5_	−4.7	4-oxododecaneoic acid	
45	7.8	340.095398	340, 296, 257, 241, 210	C_17_H_14_N_3_O_5_	−4.4	unknown	
46	8.2	551.215501	551, 341, 329, 205	C_28_H_31_N4O_8_	−1.4	unknown	
47	10.4	609.293709	609, 565, 463, 301, 113	C_31_H_45_O_12_	−3.4	unknown	
26	12.7	227.128985	227, 183, 165	C_12_H_19_O_4_	−0.5	traumatic acid	3.19 ± 0.01
27	13.0	327.217951	327, 211, 171	C_18_H_31_O_5_	−0.8	9,10-dihydroxy-8-oxooctadec-12-enoic acid	0.07 ± 0.01
28	14.2	329.233675	329, 229, 211, 171	C_18_H_33_O_5_	−1.0	pinellic acid	0.06 ± 0.01
48	15.9	307.191932	307, 235, 211, 185, 121	C_18_H_27_O_4_	−1.5	linoleic acid derivat.	
29	16.6	311.187090	311, 293, 267	C_17_H_27_O_5_	−2.2	octadecdienoic acid derivat.	
49	18.3	311.223767	311, 293, 211	C_18_H_31_O_4_	−3.2	9(S)-HPODE	
50	20.0	313.239690	313, 295, 201	C_18_H_33_O_4_	−4.0	9,10-DHOME	
51	21.1	293.213413	293, 275, 235, 183	C_18_H_29_O_3_	−4.1	linoleic acid derivat.	
52	21.3	293.213318	293, 275, 223, 195	C_18_H_29_O_3_	−3.7	linoleic acid derivat.	
53	22.5	295.228378	295, 277, 195	C_18_H_31_O_3_	−1.7	linoleic acid derivat.	
54	22.7	295.228280	295, 277, 171	C_18_H_31_O_3_	−1.4	linoleic acid derivat.	

Compounds were identified based on: Compound Crawler Bruker, Sirius 4.0.1. and confirmed by standards when available. RT—retention time, M/Z—mass to charge ratio.

**Table 2:** The compounds identified in individual fractions obtained from *C. vulgaris* methanol/water extract (mg/g ± SD of fraction).

Nr	Compounds	HCV	EACV	BCV	H_2_OCV
1	quinic acid	ND	ND	ND	17.63 ± 0.11
2	dihydroxybenzoic acid	ND	ND	5.8 ± 0.12	1.1 ± 0.02
34	L-typtophan	ND	ND	ND	+
4	neochorogenic acid	ND	23.20 ± 0.12	0.09 ± 0.01	0.06 ± 0.01
5	chlorogenic acid	ND	157.69 ± 0.12	1.32 ± 0.18	0.12 ± 0.01
35	methyltryptophan	ND	ND	ND	+
36	carlinoside	ND	57.71 ± 0.23	1.92 ± 0.12	ND
11	schaftoside	ND	29.07 ± 0.02	1.01 ± 0.10	ND
12	orientin	ND	ND	35.03 ± 0.03	ND
13	isoschaftoside	ND	71.46 ± 0.02	1.90 ± 0.12	ND
37	taxifolin	ND	1.17 ± 0.11	0.42 ± 0.06	ND
14	isoorientin	ND	ND	22.45 ± 0.12	ND
38	rutin	ND	23.88 ± 0.11	1.65 ± 0.10	ND
39	vitexin	ND	74.18 ± 0.13	2.98 ± 0.12	ND
40	apigenin di-C arabinoside	ND	0.93 ± 0.02	0.02 ± 0.01	ND
41	densifloside	ND	113.73 ± 0.26	7.89 ± 0.12	ND
42	nicotiflorin	ND	18.17 ± 0.02	2.90 ± 0.13	ND
43	azelaic acid	ND	+	+	ND
44	4-oxododecaneoic acid	ND	+	+	ND
45	NZ	ND	+	+	ND
46	NZ	ND	ND	+	ND
47	NZ	ND	+	+	ND
26	traumatic acid	ND	43.04 ± 0.05	ND	ND
27	9,10-dihydroxy-8-oxooctadec-12-enoic acid	ND	29.60 ± 0.08	ND	ND
28	pinellic acid	ND	36.57 ± 0.12	ND	ND
48	linoleic acid derivat.	ND	+	ND	ND
29	octadecdienoic acid derivat.	ND	+	ND	ND
49	9(S)-HPODE	ND	+	ND	ND
50	9,10-DHOME	ND	+	ND	ND
51	linoleic acid derivat.	+	ND	ND	ND
52	linoleic acid derivat.	+	ND	ND	ND
53	linoleic acid derivat.	+	ND	ND	ND
54	linoleic acid derivat.	+	ND	ND	ND

ND—not detected; +—detected.

**Table 3:** The results of radical scavenging activity (DPPH) and ferric reducing antioxidant power (FRAP) obtained for hexane (HCV), ethyl acetate (EaCV), butanol (BCV), and water (H_2_OCV) fractions from the methanolic extract of *Carlina vulgaris*. Values are means ± standard deviation (SD) of triplicate.

Fractions	Concentration (µg/mL)	Equivalent of Trolox Concentration (DPPH)	Equivalent of Ascorbic Acid Concentration (FRAP)
H_2_OCV	25	5.062 ± 0.698	4.581 ± 0.223
	100	14.221 ± 0.451	17.207 ± 0.632
	200	22.322 ± 0.516	34.860 ± 0.258
BCV	25	3.330 ± 0.799	2.793 ± 0.428
	100	10.429 ± 0.296	11.397 ± 0.011
	200	20.458 ± 0.309	21.676 ± 0.223
EaCV	25	10.988 ± 0.420	10.056 ± 0.365
	100	29.581 ± 0.390	39.33 ± 0.447
	200	42.992 ± 0.160	74.413 ± 1.210
HCV	25	0.081 ± 0.011	0.894 ± 0.447
	100	0.773 ± 0.535	3.017 ± 0.223
	200	1.133 ± 0.579	4.804 ± 0.223

The term “equivalent of ascorbic acid/Trolox” means that the reducing/antioxidant power of the extract at a given concentration is equivalent to the reducing power of a given concentration of ascorbic acid/Trolox.

## Data Availability

Data are contained within the article.

## References

[1] Gilca M., Tiplica G.S., Salavastru C.M. (2018). Traditional and Ethnobotanical Dermatology Practices in Romania and Other Eastern European Countries. Clin. Dermatol..

[2] Menković N., Šavikin K., Tasić S., Zdunić G., Stešević D., Milosavljević S., Vincek D. (2011). Ethnobotanical Study on Traditional Uses of Wild Medicinal Plants in Prokletije Mountains (Montenegro). J. Ethnopharmacol..

[3] Sen D.B., Kumar Sen A., Patel K.P., Balaraman R., Shah U., Maheshwari R.A. (2022). Anti-Ulcer Activities of Herbal Remedies as Alternative Therapy. JNR.

[4] Dénes A., Papp N., Babai D., Czúcz B., Molnár Z. (2012). Wild Plants Used for Food by Hungarian Ethnic Groups Living in the Carpathian Basin. Acta Soc. Bot. Pol..

[5] Bonet M.À., Parada M., Selga A., Vallès J. (1999). Studies on Pharmaceutical Ethnobotany in the Regions of L’Alt Empordà and Les Guilleries (Catalonia, Iberian Peninsula). J. Ethnopharmacol..

[6] De Natale A., Pezzatti G.B., Pollio A. (2009). Extending the Temporal Context of Ethnobotanical Databases: The Case Study of the Campania Region (Southern Italy). J. Ethnobiol. Ethnomedicine.

[7] Pieroni A., Nedelcheva A., Hajdari A., Mustafa B., Scaltriti B., Cianfaglione K., Quave C.L. (2014). Local Knowledge on Plants and Domestic Remedies in the Mountain Villages of Peshkopia (Eastern Albania). J. Mt. Sci..

[8] Kozlowska W., Wagner C., Moore E.M., Matkowski A., Komarnytsky S. (2018). Botanical Provenance of Traditional Medicines from Carpathian Mountains at the Ukrainian-Polish Border. Front. Pharmacol..

[9] Rexhepi B., Mustafa B., Hajdari A., Rushidi-Rexhepi J., Quave C.L., Pieroni A. (2013). Traditional Medicinal Plant Knowledge among Albanians, Macedonians and Gorani in the Sharr Mountains (Republic of Macedonia). Genet. Resour. Crop Evol..

[10] Guarrera P.M. (2003). Food Medicine and Minor Nourishment in the Folk Traditions of Central Italy (Marche, Abruzzo and Latium). Fitoterapia.

[11] Strzemski M., Wójciak-Kosior M., Sowa I., Załuski D., Verpoorte R. (2019). Historical and Traditional Medical Applications of *Carlina Acaulis* L.—A Critical Ethnopharmacological Review. J. Ethnopharmacol..

[12] Strzemski M., Wojnicki K., Sowa I., Wojas-Krawczyk K., Krawczyk P., Kocjan R., Such J., Latalski M., Wnorowski A., Wójciak-Kosior M. (2017). In Vitro Antiproliferative Activity of Extracts of Carlina Acaulis Subsp. Caulescens and Carlina Acanthifolia Subsp. Utzka. Front. Pharmacol..

[13] Spinozzi E., Ferrati M., Cappellacci L., Caselli A., Perinelli D.R., Bonacucina G., Maggi F., Strzemski M., Petrelli R., Pavela R. (2023). *Carlina Acaulis* L. (Asteraceae): Biology, Phytochemistry, and Application as a Promising Source of Effective Green Insecticides and Acaricides. Ind. Crops Prod..

[14] Wnorowska S., Targowska-Duda K., Kurzepa J., Wnorowski A., Strzemski M. (2022). Carlina Oxide Inhibits the Interaction of SARS-CoV-2 S Glycoprotein with Angiotensin-Converting Enzyme 2. Ind. Crops Prod..

[15] Wnorowski A., Wnorowska S., Wojas-Krawczyk K., Grenda A., Staniak M., Michalak A., Woźniak S., Matosiuk D., Biała G., Wójciak M. (2020). Toxicity of Carlina Oxide—A Natural Polyacetylene from the Carlina Acaulis Roots—In Vitro and in Vivo Study. Toxins.

[16] Rosato A., Barbarossa A., Mustafa A.M., Bonacucina G., Perinelli D.R., Petrelli R., Maggi F., Spinozzi E. (2021). Comprehensive Evaluation of the Antibacterial and Antifungal Activities of *Carlina Acaulis* L. Essential Oil and Its Nanoemulsion. Antibiotics.

[17] Konechna R., Khropot O., Petrina R., Kurka M., Gubriy Z., Novikov V. (2017). Research of Antioxidant Properties Of Extracts Of The Plants And The Callus Biomass. Asian J. Pharm. Clin. Res..

[18] Link P., Roth K., Sporer F., Wink M. (2016). Carlina Acaulis Exhibits Antioxidant Activity and Counteracts Aβ Toxicity in Caenorhabditis Elegans. Molecules.

[19] Stojanović-Radić Z., Čomić L., Radulović N., Blagojević P., Mihajilov-Krstev T., Rajković J. (2012). Commercial *Carlinae Radix* Herbal Drug: Botanical Identity, Chemical Composition and Antimicrobial Properties. Pharm. Biol..

[20] Lunz K., Stappen I. (2021). Back to the Roots—An Overview of the Chemical Composition and Bioactivity of Selected Root-Essential Oils. Molecules.

[21] Kavallieratos N.G., Nika E.P., Skourti A., Spinozzi E., Ferrati M., Petrelli R., Maggi F., Benelli G. (2022). Carlina Acaulis Essential Oil: A Candidate Product for Agrochemical Industry Due to Its Pesticidal Capacity. Ind. Crops Prod..

[22] Dresler S., Hawrylak-Nowak B., Strzemski M., Wójciak-Kosior M., Sowa I., Hanaka A., Gołoś I., Skalska-Kamińska A., Cieślak M., Kováčik J. (2019). Metabolic Changes Induced by Silver Ions in Carlina Acaulis. Plants.

[23] Strzemski M., Wójciak-Kosior M., Sowa I., Rutkowska E., Szwerc W., Kocjan R., Latalski M. (2016). Carlina Species as a New Source of Bioactive Pentacyclic Triterpenes. Ind. Crops Prod..

[24] Strzemski M., Płachno B.J., Mazurek B., Kozłowska W., Sowa I., Lustofin K., Załuski D., Rydzik Ł., Szczepanek D., Sawicki J. (2020). Morphological, Anatomical, and Phytochemical Studies of *Carlina Acaulis* L. Cypsela. IJMS.

[25] Dresler S., Strzemski M., Kováčik J., Sawicki J., Staniak M., Wójciak M., Sowa I., Hawrylak-Nowak B. (2020). Tolerance of Facultative Metallophyte Carlina Acaulis to Cadmium Relies on Chelating and Antioxidative Metabolites. IJMS.

[26] Becker U., Colling G., Dostal P., Jakobsson A., Matthies D. (2006). Local Adaptation in the Monocarpic Perennial Carlina Vulgaris at Different Spatial Scales across Europe. Oecologia.

[27] Meusel H., Kästner A., Merxmüller H., Rechinger K.H. (1994). Lebensgeschichte der Gold- und Silberdisteln. 2: Artenvielfalt und Stammesgeschichte der Gattung: Zum Gedächtnis an Hermann Merxmüller und für Karl-Heinz Rechinger; Denkschriften/Österreichische Akademie der Wissenschaften, Mathematisch-Naturwissenschaftliche Klasse.

[28] Strzemski M., Wójciak-Kosior M., Sowa I., Załuski D., Szwerc W., Sawicki J., Kocjan R., Feldo M., Dresler S. (2017). *Carlina Vulgaris* L. as a Source of Phytochemicals with Antioxidant Activity. Oxidative Med. Cell. Longev..

[29] Belabbes R., Mami I.R., Dib M.E.A., Mejdoub K., Tabti B., Costa J., Muselli A. (2020). Chemical Composition and Biological Activities of Essential Oils of Echinops Spinosus and Carlina Vulgaris Rich in Polyacetylene Compounds. CNF.

[30] Herrmann F., Hamoud R., Sporer F., Tahrani A., Wink M. (2011). Carlina Oxide—A Natural Polyacetylene from *Carlina Acaulis* (Asteraceae) with Potent Antitrypanosomal and Antimicrobial Properties. Planta Med..

[31] Xiao J., Capanoglu E., Jassbi A.R., Miron A. (2016). Advance on the Flavonoid *C*-Glycosides and Health Benefits. Crit. Rev. Food Sci. Nutr..

[32] Xie L., Deng Z., Zhang J., Dong H., Wang W., Xing B., Liu X. (2022). Comparison of Flavonoid O-Glycoside, C-Glycoside and Their Aglycones on Antioxidant Capacity and Metabolism during In Vitro Digestion and In Vivo. Foods.

[33] Courts F.L., Williamson G. (2015). The Occurrence, Fate and Biological Activities of *C* -Glycosyl Flavonoids in the Human Diet. Crit. Rev. Food Sci. Nutr..

[34] Wen L., Zhao Y., Jiang Y., Yu L., Zeng X., Yang J., Tian M., Liu H., Yang B. (2017). Identification of a Flavonoid C -Glycoside as Potent Antioxidant. Free. Radic. Biol. Med..

[35] Choi J.S., Islam M.N., Ali M.Y., Kim Y.M., Park H.J., Sohn H.S., Jung H.A. (2014). The Effects of C-Glycosylation of Luteolin on Its Antioxidant, Anti-Alzheimer’s Disease, Anti-Diabetic, and Anti-Inflammatory Activities. Arch. Pharm. Res..

[36] Gomes A.C.C., Sampaio L.D.S., Silva P.A.D., Lamas M.E., Sakuragui C.M., Barreto Junior C.B., Simas N.K., Kuster R.M. (2014). In Vitro effect of isoschaftoside isolated from *Syngonium Podophyllum* on pig kidney Na^+^, K^+^-ATPASE. Química Nova.

[37] Guan S., Sun L., Wang X., Huang X., Luo T. (2022). Isoschaftoside Inhibits Lipopolysaccharide-Induced Inflammation in Microglia through Regulation of HIF-1α-Mediated Metabolic Reprogramming. Evid.-Based Complement. Altern. Med..

[38] Chen Y.-L., Chen C.-Y., Lai K.-H., Chang Y.-C., Hwang T.-L. (2023). Anti-Inflammatory and Antiviral Activities of Flavone C-Glycosides of Lophatherum Gracile for COVID-19. J. Funct. Foods.

[39] Raynaud J., Rasolojaona L. (1979). Flavonoïdes Des Feuilles de Carlina Acaulis. Planta Med..

[40] Dordević S., Tadić V., Petrović S., Kukić-Marković J., Dobrić S., Milenković M., Hadžifejzović N. (2012). Bioactivity Assays on Carlina Acaulis and C. Acanthifolia Root and Herb Extracts. Dig. J. Nanomater. Biostructures.

[41] Rathod N.B., Elabed N., Punia S., Ozogul F., Kim S.-K., Rocha J.M. (2023). Recent Developments in Polyphenol Applications on Human Health: A Review with Current Knowledge. Plants.

[42] Zhang H., Tsao R. (2016). Dietary Polyphenols, Oxidative Stress and Antioxidant and Anti-Inflammatory Effects. Curr. Opin. Food Sci..

[43] Santana-Gálvez J., Cisneros-Zevallos L., Jacobo-Velázquez D. (2017). Chlorogenic Acid: Recent Advances on Its Dual Role as a Food Additive and a Nutraceutical against Metabolic Syndrome. Molecules.

[44] Liang N., Kitts D. (2015). Role of Chlorogenic Acids in Controlling Oxidative and Inflammatory Stress Conditions. Nutrients.

[45] Lee K.-H., Do H.-K., Kim D.-Y., Kim W. (2021). Impact of Chlorogenic Acid on Modulation of Significant Genes in Dermal Fibroblasts and Epidermal Keratinocytes. Biochem. Biophys. Res. Commun..

[46] Zada S., Pham T.M., Hwang J.S., Ahmed M., Lai T.H., Elashkar O., Kim J.-H., Kim D.H., Kim D.R. (2021). Chlorogenic Acid Protects Human Chondrocyte C28/I2 Cells from Oxidative Stress-Induced Cell Death through Activation of Autophagy. Life Sci..

[47] Babaei F., Moafizad A., Darvishvand Z., Mirzababaei M., Hosseinzadeh H., Nassiri-Asl M. (2020). Review of the Effects of Vitexin in Oxidative Stress-related Diseases. Food Sci. Nutr..

[48] An F., Yang G., Tian J., Wang S. (2012). Antioxidant Effects of the Orientin and Vitexin in Trollius Chinensis Bunge in D-Galactose-Aged Mice. Neural Regen. Res..

[49] Attiq A., Jalil J., Husain K., Mohamad H.F., Ahmad A. (2021). Luteolin and Apigenin Derived Glycosides from Alphonsea Elliptica Abrogate LPS-Induced Inflammatory Responses in Human Plasma. J. Ethnopharmacol..

[50] Lee J.H., Park J., Shin D.W. (2022). The Molecular Mechanism of Polyphenols with Anti-Aging Activity in Aged Human Dermal Fibroblasts. Molecules.

[51] Khalid K.A., Nawi A.F.M., Zulkifli N., Barkat M.A., Hadi H. (2022). Aging and Wound Healing of the Skin: A Review of Clinical and Pathophysiological Hallmarks. Life.

[52] Shin J.-W., Kwon S.-H., Choi J.-Y., Na J.-I., Huh C.-H., Choi H.-R., Park K.-C. (2019). Molecular Mechanisms of Dermal Aging and Antiaging Approaches. IJMS.

[53] Strzemski M., Dzida K., Dresler S., Sowa I., Kurzepa J., Szymczak G., Wójciak M. (2021). Nitrogen Fertilisation Decreases the Yield of Bioactive Compounds in *Carlina Acaulis* L. Grown in the Field. Ind. Crops Prod..

[54] Ziemlewska A., Nizioł-Łukaszewska Z., Bujak T., Zagórska-Dziok M., Wójciak M., Sowa I. (2021). Effect of Fermentation Time on the Content of Bioactive Compounds with Cosmetic and Dermatological Properties in Kombucha Yerba Mate Extracts. Sci. Rep..

[55] Sowa I., Paduch R., Strzemski M., Zielińska S., Rydzik-Strzemska E., Sawicki J., Kocjan R., Polkowski J., Matkowski A., Latalski M. (2018). Proliferative and Antioxidant Activity of *Symphytum Officinale* Root Extract. Nat. Prod. Res..

[56] Wójciak M., Feldo M., Borowski G., Kubrak T., Płachno B.J., Sowa I. (2022). Antioxidant Potential of Diosmin and Diosmetin against Oxidative Stress in Endothelial Cells. Molecules.

[57] Ziemlewska A., Zagórska-Dziok M., Nizioł-Łukaszewska Z., Kielar P., Mołoń M., Szczepanek D., Sowa I., Wójciak M. (2023). In Vitro Evaluation of Antioxidant and Protective Potential of Kombucha-Fermented Black Berry Extracts against H2O2-Induced Oxidative Stress in Human Skin Cells and Yeast Model. IJMS.

